# Extensor Pollicis Longus Entrapment in a Pediatric Distal Radius Fracture: A Case Report and Review of the Literature

**DOI:** 10.7759/cureus.84875

**Published:** 2025-05-27

**Authors:** Fahad Abduljabbar

**Affiliations:** 1 Orthopedic Surgery, King Abdulaziz University Hospital, Jeddah, SAU

**Keywords:** distal radius fracture, entrapment, extensor pollicis longus, tendon, tenolysis

## Abstract

Entrapment of the extensor pollicis longus (EPL) tendon following a pediatric distal radius fracture is an exceptionally rare occurrence that can clinically mimic tendon rupture. We report the case of a 12-year-old girl who presented with loss of active thumb extension several weeks after successful reduction of a distal radius fracture. Imaging studies ruled out tendon rupture, and subsequent surgical exploration confirmed entrapment of the EPL within the fracture callus. The patient underwent tenolysis and achieved good functional recovery. This case highlights the importance of considering tendon entrapment in patients with unexplained thumb dysfunction following forearm fractures.

## Introduction

Distal radius fractures are among the most common injuries encountered in the pediatric population and typically result from falls onto an outstretched hand. These fractures are usually managed conservatively with closed reduction and immobilization, yielding favorable outcomes in most cases [1]. A recognized complication associated with distal radius fractures is injury to the extensor pollicis longus (EPL) tendon, which frequently presents as a delayed rupture caused by mechanical attrition, ischemia, or entrapment [2,3]. Although rupture of the EPL tendon is relatively rare in children, EPL tendon entrapment at the fracture site represents an unusual and underreported phenomenon, with only five cases documented in the literature [3-7].

The mechanism underlying EPL entrapment involves the tendon being drawn into the fracture site [4,5], particularly in cases involving volar displacement or forceful wrist movements during injury or reduction, leading to incarceration between fracture fragments [4,5]. In such scenarios, sharp fracture edges or displaced bone fragments can act as a trap for the EPL tendon, especially when the wrist is acutely flexed or extended. This can result in the tendon being pinched or incarcerated during the initial trauma or during attempted closed reduction, restricting its gliding motion and ultimately leading to entrapment within the healing callus. Entrapment can present acutely, often with difficulty in achieving fracture reduction, or more commonly as a delayed loss of thumb extension due to the tendon becoming tethered within the ossifying callus [8]. Because the clinical presentation may mimic tendon rupture, there is a risk of misdiagnosis and, consequently, inappropriate management.

Awareness of this rare condition is essential to prevent functional impairment. In this report, we present a pediatric case of delayed EPL tendon entrapment within the fracture callus of a volarly displaced distal radius fracture. Emphasis is placed on the diagnostic approach, surgical management, and a review of relevant literature to underscore the importance of considering this diagnosis in patients presenting with post-injury thumb extension deficits.

## Case presentation

**Figure 1:**
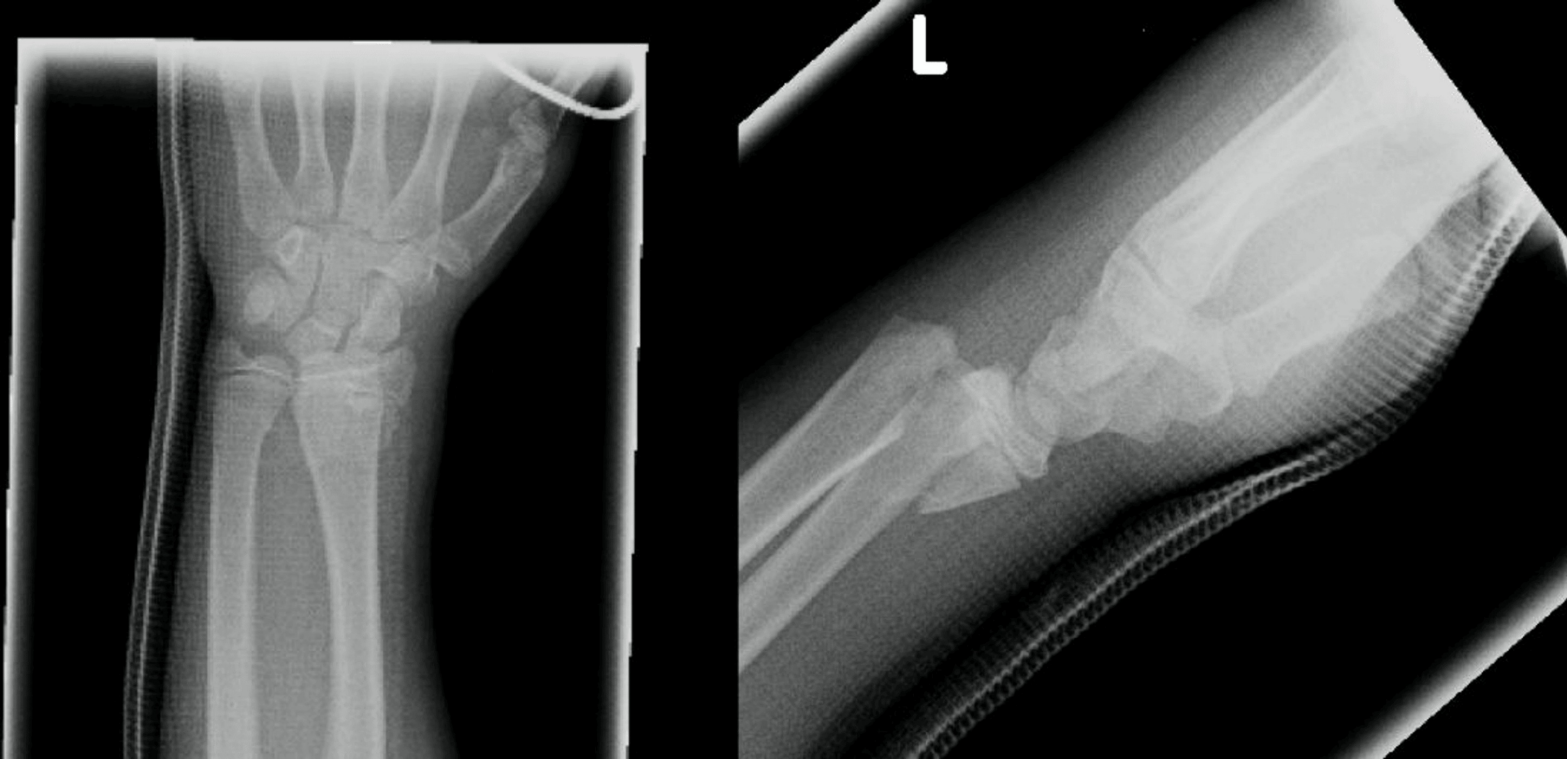
Anteroposterior (AP) and lateral radiographs of the left wrist demonstrating a volarly displaced distal radius fracture.

**Figure 2:**
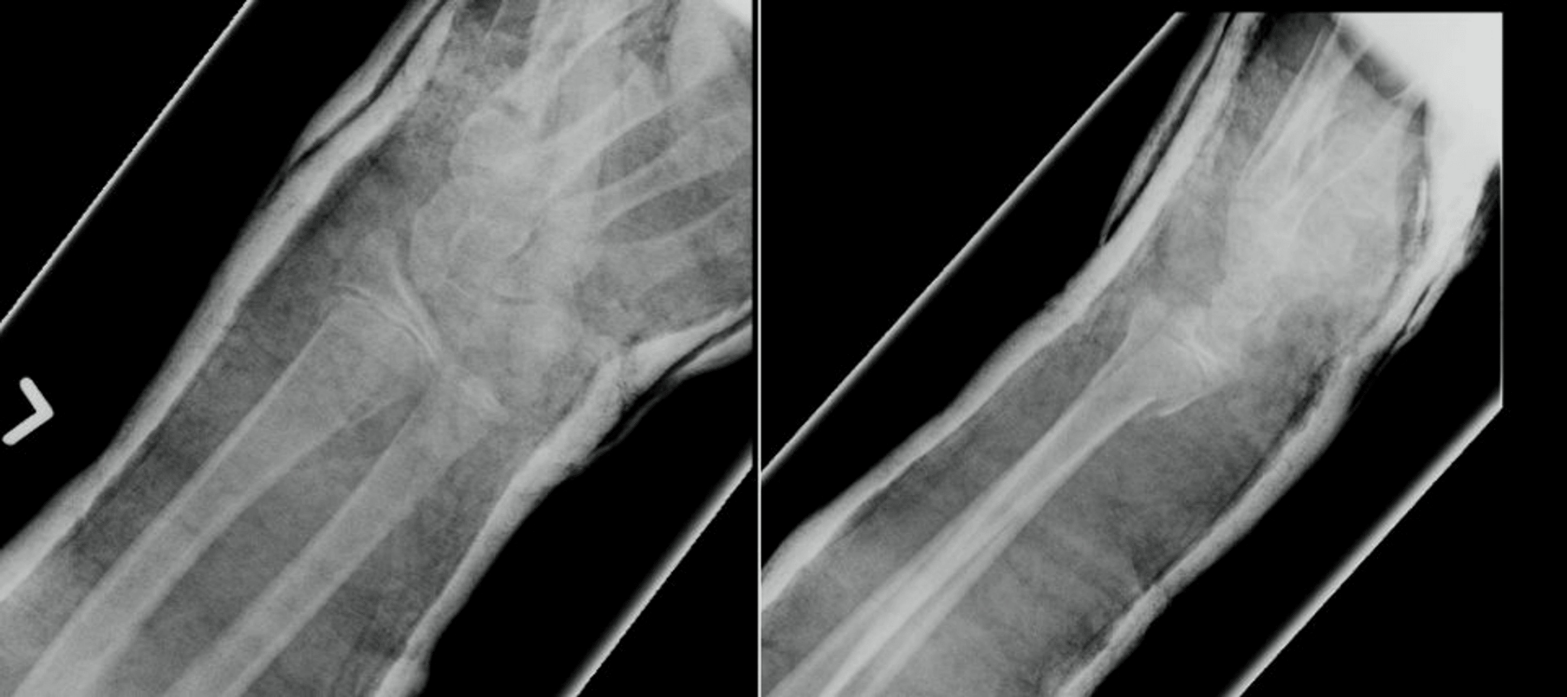
Anteroposterior (AP) and lateral radiographs after reduction and casting, showing satisfactory alignment.

A 12-year-old girl presented with a closed type II Salter-Harris fracture of the left distal radius (Figure 1). The emergency team performed closed reduction and cast immobilization (Figure 2).

She was referred to the orthopedic outpatient clinic for follow-up 10 days later. At her initial visit, she had normal active thumb extension and a normal neurovascular examination, including assessment of the posterior interosseous nerve (PIN). Repeat wrist radiographs demonstrated satisfactory alignment. At her second follow-up visit, four weeks post-injury, she remained immobilized. Physical examination revealed a loss of active thumb extension, suggestive of absent EPL function and raising suspicion of tendon rupture. Her neurovascular status remained intact.

**Figure 3:**
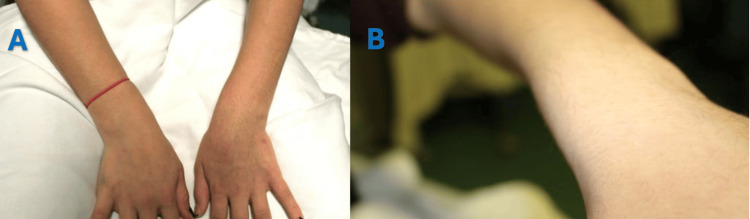
(A) Clinical photographs of both wrists showing a dorsal prominence in the left wrist secondary to callus formation; (B) Clinical photograph of the left wrist demonstrating a pronounced dorsal prominence.

**Figure 4:**
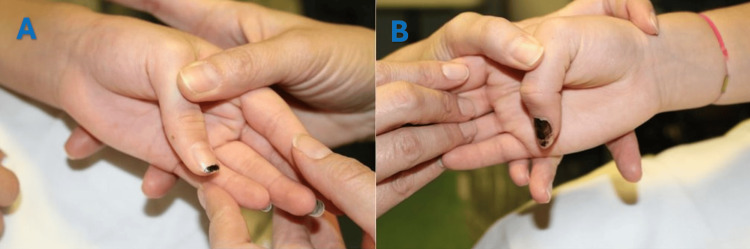
(A) Clinical photograph showing normal tenodesis effect of the right extensor pollicis longus tendon; (B) Clinical photograph showing loss of tenodesis effect of the left extensor pollicis longus tendon.

USG, however, demonstrated that the EPL tendon was intact both proximal and distal to the fracture site. Further clinical examination at six weeks post-injury revealed abundant callus formation over the dorsoradial aspect of the wrist (Figure 3), accompanied by a loss of the tenodesis effect of the left thumb (Figure 4).

**Figure 5:**
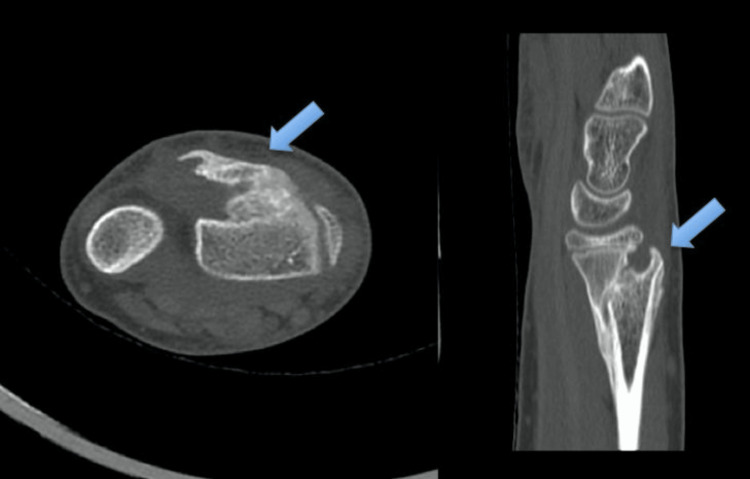
CT scan of the left wrist showing axial and sagittal cuts demonstrating the dorsal bridging callus (blue arrow) responsible for extensor pollicis longus tendon entrapment.

**Figure 6:**
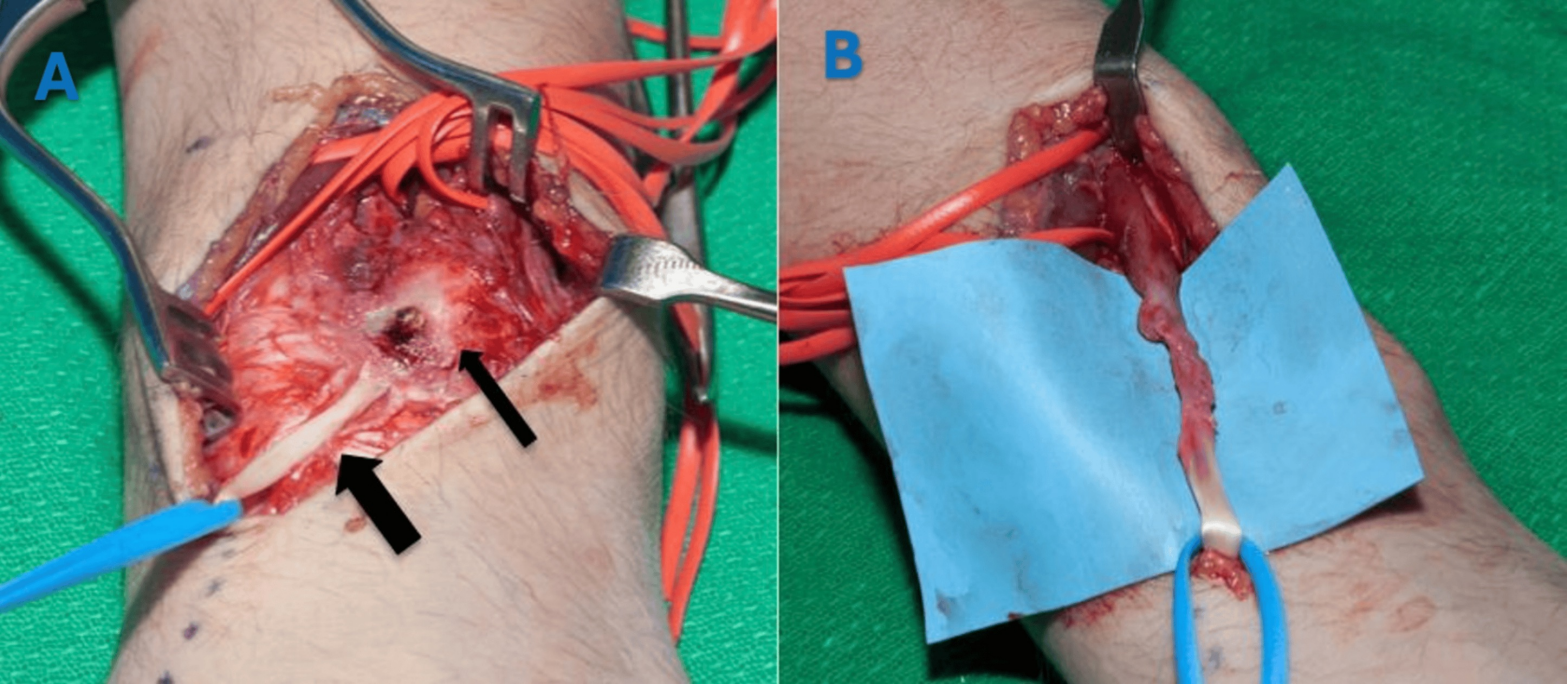
(A) Intraoperative photograph showing the entrapped extensor pollicis longus (EPL) tendon (large arrow) within the fracture callus (small arrow) at the level of the osseous beak. (B) EPL tendon following tenolysis.

A standard dorsal approach to the wrist was utilized to access the EPL tendon. A longitudinal incision was made over the dorsoradial aspect of the wrist, centered on Lister’s tubercle, allowing exposure of the third extensor compartment. Dissection was carried through the subcutaneous tissue and extensor retinaculum to identify the EPL tendon proximally. This approach enabled direct visualization of the callus formation encasing the tendon, allowing for precise and controlled tenolysis. Intraoperatively, the EPL tendon was found entrapped (engulfed) within the fracture callus at the osseous beak (Figures 5-6).

Tenolysis was performed using an osteotome and rongeur. The tendon was first identified proximal to the fracture site and carefully dissected at the level of the callus. Under fluoroscopic guidance, the callus was meticulously removed until the tendon became visible distally, without any iatrogenic damage. One of the key challenges in such cases is to release the entrapped tendon from the callus without inflicting injury, a goal that was achieved here through meticulous dissection. Additionally, the tendon was transposed radially to Lister’s tubercle. Passive intraoperative range of motion (ROM) testing of the thumb’s interphalangeal joint confirmed that the EPL tendon glided freely without residual entrapment. Postoperatively, the patient was provided with a protective removable wrist splint for two weeks, and active ROM exercises were encouraged. At the two-year final follow-up, the patient exhibited a loss of 5° of active thumb extension compared to the unaffected right thumb, without any functional limitations. Grip strength testing revealed slightly reduced strength in the left hand (50.9 lbs) compared to the right hand (56.8 lbs).

## Discussion

In this case report, we present an instance of EPL tendon entrapment within the fracture callus following a distal radius fracture in a pediatric patient. The EPL originates from the posterior surface of the ulna and the interosseous membrane, passing around Lister’s tubercle at the distal radius before inserting at the base of the distal phalanx of the thumb. Due to its anatomical course, particularly as it passes through the third extensor compartment, the EPL is particularly vulnerable to injury or entrapment during distal radius fractures. Other extensor tendons, such as the extensor digitorum communis (EDC) and extensor indicis proprius (EIP), lie in adjacent compartments and may also be affected depending on the fracture pattern and callus formation. Complications involving the EPL tendon following distal radius fractures in adults include entrapment, irritation, or rupture [2]. Although EPL tendon rupture is uncommon, it has been documented in the literature as a complication following pediatric distal radius fractures [9]. However, EPL entrapment following pediatric distal radius fractures has rarely been reported (Table 1) [4-7]. EPL tendon rupture associated with Colles’ fractures typically occurs late and most often in non-displaced fractures [10]. Trevor D et al. [11] suggested avascular necrosis as the primary cause of EPL tendon rupture in dorsally displaced fractures. Conversely, in volarly displaced fractures, the mechanism differs significantly: extreme wrist flexion stretches the musculotendinous unit, and the sharp dorsal edge of the proximal fragment can tear the tendon or entrap it within the fracture site [4].

**Table 1:** Reported cases of EPL entrapment in the pediatric literature. L: Left; R: Right; DR: Distal radius; SH: Salter-Harris; ORIF: Open reduction and internal fixation; CRPP: Closed reduction and percutaneous pinning. *Both volar and dorsal incisions were used.

Author, Year	Age, Sex	Fracture Side/Type	Mechanism of Injury	Time of EPL Entrapment Diagnosis	Intervention	Outcome
Cavanilles Walker JM et al., (2012) [3]	6M	L distal radius and ulna (displaced dorsally)	Fall onto outstretched hand	5 months post-CRPP	EIP to EPL tendon transfer	Full range of motion and good function
Uchida Y and Sugioka Y (1990) [4]	14M	R distal radius SH II (displaced volarly)	Forced hyperflexion of the wrist	6 weeks post-closed reduction	EPL tendon reconstruction with PL/plantaris	Good function with some limitation of thumb IP flexion
Thomas WG and Kershaw CJ (1988) [5]	13F	L distal radius SH II (displaced volarly)	Fall from bicycle on L hand	At presentation	ORIF* as closed reduction failed / EPL tenolysis	Good recovery
El-Kazzi W and Schuind F (2005) [6]	12M	R distal radius (displaced volarly)	Fall from bicycle	6 weeks	EPL tendon reconstruction with PL	Full range of motion and good function
Mansour AA 3rd, et al. (2013) [7]	9M	R distal radius fracture (displaced volarly)	Fall from scooter	8 weeks	Tenolysis	Full return to activity without limitation
Current study	12F	L distal radius fracture SH II (displaced volarly)	Fall onto outstretched hand	6 weeks	Tenolysis	Full recovery without limitation

Entrapment of the EPL tendon most commonly occurs dorsally, particularly in the region just proximal to Lister’s tubercle, where the tendon is closest to the radius. Callus formation at this location can tether the tendon within the healing bone, resulting in delayed loss of thumb extension. In contrast, entrapment occurring more ulnarly may involve the EIP, potentially mimicking or affecting index finger extension. Volar callus entrapment is less commonly reported but may implicate flexor tendons, leading to different clinical manifestations such as reduced thumb or finger flexion. The mechanism of EPL tendon entrapment following distal radius fractures is primarily attributed to volar displacement combined with forceful supination of the distal fragment, which displaces the EPL tendon toward the ulnar side and interposes it between the fracture fragments. This may ultimately lead to tendon rupture [12]. EPL entrapment is most commonly associated with volarly displaced distal radius fractures. Four of the five previously reported cases of EPL entrapment following distal radius fractures involved volar displacement [4-7]. All these cases involved entrapment at the site of callus formation, which explains why the patients initially had normal EPL function, as callus typically takes about 4 to 6 weeks to form around the fracture edges. In contrast to our patient, only one previously documented case involved a dorsally displaced fracture, which was notably diagnosed late, approximately five months post-injury [3].

Clinically, EPL tendon entrapment may present acutely as an irreducible fracture due to tendon interposition between fracture fragments, necessitating early surgical exploration [6]. Delayed presentations typically include loss of thumb extension, as observed in our patient, dorsal wrist pain along the course of the EPL, and a diminished tenodesis effect with wrist flexion [8]. Due to its clinical similarity, EPL entrapment is often misdiagnosed as a tendon rupture.

The diagnosis of EPL entrapment requires a high index of suspicion, particularly in cases of persistent dorsal wrist pain or loss of thumb function following a distal radius fracture. Mansour AA et al. suggested that displacement of the dorsal metaphyseal cortex may serve as a radiographic clue indicative of potential EPL tendon entrapment [7]. To date, there are no clear diagnostic guidelines for EPL entrapment. CT scan, MRI, or ultrasound may help delineate EPL anatomy and differentiate tendon rupture from entrapment. However, surgical exploration should not be delayed in the presence of thumb dysfunction. Tenolysis is considered the first-line treatment for EPL entrapment [6]. If tenolysis is not feasible, alternative surgical options include EIP tendon transfer or tendon reconstruction using the palmaris longus tendon [3,5,13]. Of the five previously reported cases, two were successfully managed with tenolysis [5,7], while the remaining three required either tendon transfer or reconstruction [3].

## Conclusions

Entrapment of the EPL tendon following pediatric distal radius fractures is exceptionally rare but clinically significant, as it can closely mimic tendon rupture, particularly when thumb extension is unexpectedly lost in the weeks following injury. This case highlights the importance of maintaining a high index of suspicion for tendon entrapment in patients who develop unexpected functional deficits during follow-up. Early use of imaging modalities such as ultrasound or CT, combined with careful clinical evaluation, can effectively distinguish between tendon rupture and entrapment. Prompt surgical intervention, when indicated, typically results in excellent functional recovery, as demonstrated by our patient’s outcome. Clinicians should remain alert to this uncommon complication, and a high index of suspicion is key to ensuring proper diagnosis and management, preventing diagnostic delays, and optimizing patient outcomes.
